# Perception versus preference: The role of self-assessed risk measures on individual mitigation behaviors during the COVID-19 pandemic

**DOI:** 10.1371/journal.pone.0254756

**Published:** 2021-08-04

**Authors:** Bachir Kassas, Stephen N. Morgan, John H. Lai, Jaclyn D. Kropp, Zhifeng Gao

**Affiliations:** 1 Food and Resource Economics Department, University of Florida, Gainesville, Florida, United States of America; 2 Economic Research Service, United States Department of Agriculture, Kansas City, Missouri, United States of America; George Washington University, UNITED STATES

## Abstract

In the midst of a global pandemic, prevention methods stand as a crucial first step toward addressing the public health crisis and controlling the spread of the virus. However, slowing the spread of the virus hinges on the public’s willingness to follow a combination of mitigation practices to avoid contracting and transmitting the disease. In this study, we investigate the factors related to individuals’ risk perceptions associated with COVID-19 as well as their general self-assessed risk preferences. We also provide insights regarding the role of risk perceptions and preferences on mitigation behavior by examining the correlation between these risk measures and both the likelihood of following various mitigation practices and total number of practices followed. Although we find both risk perceptions and preferences to be significantly correlated with mitigation behaviors, risk perceptions are correlated with a larger number of practices. Additionally, we find significant heterogeneity in mitigation behaviors across numerous individual and household characteristics. These results can serve as a benchmark for the design and development of interventions to increase awareness and promote higher adoption of mitigation practices.

## Introduction

The emergence of the 2019 novel coronavirus (COVID-19) touched off a public health emergency in the United States that significantly altered the landscape in which individuals make decisions and conduct normal daily activities. During March and April 2020, all 50 states declared a state of emergency; 49 states closed schools, bars, and in-person dining; and 45 states closed all non-essential businesses [1]. At the same time, public health officials recommended individuals adopt practices aimed at reducing the transmission of the virus. These recommendations included increased handwashing and sanitation practices, wearing face coverings (masks), and social distancing that involves staying six feet away from individuals who live outside of a person’s household [2]. Despite the public good nature of these recommendations, there has been significant heterogeneity in the adoption of mitigation practices among individuals. This heterogeneity presents an opportunity to gain a greater understanding of how individuals’ risk perceptions and risk preferences correlate with risk mitigation behaviors.

The primary objective of this study is to investigate how an individual’s risk perceptions and risk preferences relate to their use of mitigation practices during the COVID-19 pandemic. Risk perceptions are the intuitive judgments people make when they are evaluating hazards associated with some activity or technology [3]. Key components of this judgment process are how an individual labels a situation (e.g., low/high risk) and how an individual assesses the probability of a hazard event occurring [4]. Conversely, an individual’s risk preference is their consistent attitude or predisposition to risk in their decision-making. While individuals entered into the pandemic with a given set of risk attitudes and preferences, their perceptions of the risks associated with COVID-19 likely changed over time. For example, as researchers and public health officials released more information about the infection rate, symptoms, and health outcomes of the virus, individuals were likely updating their beliefs about the associated risks and the need to adopt practices to abate disease transmission. We would expect individuals who are more risk averse and who perceived more risks associated with COVID-19 to be more likely to adopt mitigation behaviors. However, little is known about the extent to which risk preferences and risk perceptions are associated with this decision, which may affect public health policy.

This article makes two important contributions to the literature. First, using nationally representative survey data, we measure risk perceptions and preferences in the context of a pandemic using two measures of risk perceptions (perceived probability of contracting COVID-19 and the number of risky places constructed as the sum of the places that the respondent deems as having a positive risk of contracting the virus) and one measure of risk preferences (self-assessed risk aversion), and examine the factors that correlate with these measures. While previous health-related research focused on measuring risk perceptions during the 2009 H1N1 flu pandemic [5, 6], these studies did not include measurements of risk preferences. Second, by using the three different risk measures, we present an approach to disentangling the multi-dimensional nature of risk by examining how risk perceptions and risk preferences differentially correlate with an individual’s adoption of COVID-19 mitigation practices. While this approach has been applied to consumer acceptance and consumption of foods with associated health risks [7–9], this decomposition, to our knowledge, has not been applied to other health-related behaviors like disease mitigation.

## Literature review

Individual behavior under risk depends on both individual risk perceptions and risk preferences [10, 11]. Risk perceptions can be influenced by many factors, including demographic characteristics (e.g., gender, income), experiences, sources of information, and trust in information sources [12]. In the context of expected utility theory, risk preferences describe the curvature of the decision-maker’s utility function over lottery outcomes [10] and represent the decision maker’s general attitude toward risk. Individual risk perceptions are broadly considered more malleable over time compared to risk preferences that tend to be more consistent.

Our study examines the extent to which risk perceptions and risk preferences correlate with household mitigation behavior in the context of the coronavirus pandemic. We primarily draw on two threads of the broader risk and decision-making literature: 1) how are risk perceptions and preferences measured, and 2) do risk perceptions and preferences correlate differentially with individual behaviors under risk?

### Measuring risk perceptions and risk preferences

In this article, we draw on three different risk measures to provide insights on individual mitigation behaviors in response to COVID-19 and examine the factors that correlate with these risk measures. The first two measures involve individual risk perceptions, while the final measure involves risk preferences.

Risk perceptions can be elicited by asking individuals whether or not they agree with different dimensions of a risky activity [7–9, 13]. Lusk and Coble [8] use four questions related to genetically modified (GM) food production and consumption with responses on a nine-point scale to measure consumer risk perceptions. This procedure can provide multi-dimensional context to risk perceptions in a given domain; however, it is ultimately a proxy for individual assessments of the probability of a hazardous event. Building on this approach, we focus on individual perceptions of exposure to COVID-19 for 14 different locations or activities (locations/activities) and use the total number of locations where individuals perceive exposure to COVID-19 as likely to measure risk perceptions in a pandemic.

Another more direct alternative is to ask individuals about the perceived probability they assign to a specific outcome. Measuring subjective risk perceptions is difficult because self-reported probabilities can be biased (non-linearly) with the objective probability of an event. When hazards have a very low probability of occurring, individuals may underestimate the probability of occurrence [14]. For example, Hayes et al. [15] find that a majority of subjects underestimate the risk of illness due to five different foodborne pathogens compared to reported illness rates. At higher probability thresholds, there is evidence that individuals may overestimate risk [16]. Taken together, these findings imply bimodal distributions of individuals between those who ignore low-probability events and those who over-estimate them [17]. In this article, we directly elicit individual perceptions of risk associated with COVID-19 by asking individuals to assess the probability that a member of their household (including themselves) would contract COVID-19. We include this measure due to the rapidly evolving nature of the pandemic and assumption that individual behaviors are likely driven by the subjective (perceived) rather than the objective probability of contracting the disease.

Risk preferences have been measured in two main ways in prior research. The first approach asks individuals to rank their assessment of their own behavior on a continuum ranging from risk averse to risk loving [18]. For example, the German Socio-Economic Panel (SOEP) survey elicits risk preferences using an 11-point Likert scale [19]. This approach can also be framed generally to capture a single stable risk preference across activities or directly contextualized over specific risk attitudes in a given decision domain like health or financial decision-making [20]. In this study, we use a generic 11-point scale and ask individuals to characterize themselves as someone who avoids risk (risk averse) or someone who likes to take risks (risk loving). We use a general risk measurement framing for two main reasons. First, the question is easy for subjects to understand. Second, the impacts of COVID-19 span multiple domains (e.g., financial, health, education, etc.) and do not fit into a single domain-specific category.

The second approach to assessing risk preferences asks individuals to make choices over lotteries in incentive compatible environments [18]. We do not adopt this approach because of the uncertain nature of the disease and the lack of clear probabilities with which to frame individual outcomes. Additionally, unlike lotteries, the potential outcomes of infection are difficult to capture in terms of a monetary payout.

### Effects of risk perceptions and preferences on behavior

Existing literature on risk perceptions and behaviors in a pandemic is relatively thin. In the context of the H1N1 flu pandemic, Seale et al. [5] assess risk perceptions and adoption of mitigation behaviors (e.g., hand washing, avoiding crowds, reduced social contact, etc.) among consumers in Australia. The authors find individuals who perceive the probability of contracting H1N1 to be high or very high are 2.8 times more likely to modify their behaviors than other subjects. Their study provides strong evidence that risk perceptions can change pandemic-related mitigation behaviors, but does not include a measure of risk preferences. Other studies document the changes in individual perceptions and intended behaviors but not the changes in observed behavior due to the H1N1 pandemic. Lee et al. [6] show that exposure to an individual sneezing in public significantly increases risk perceptions across a variety of related and unrelated health threats. Gidengil, Parker, and Zikmund-Fisher [21] find that risk perceptions track objective viral infections by increasing initially and then decreasing throughout the pandemic. However, increased risk perceptions are associated with increased intention to be vaccinated. Focusing on hypothetical flu scenarios, Rubinstein et al. [22] find more severe framing increases individual acceptance of medical recommendations on vaccines and antiviral medication among focus groups in England.

Outside of the pandemic framing, there is significant evidence that perceptions and preferences correlate with other types of health behavior. For instance, Brewer et al. [23] find that individuals with higher perceptions of risk are more likely to be vaccinated against Lyme disease and, once vaccinated, report reduced risk perceptions. Similar findings show higher risk perceptions are associated with higher vaccination rates in a meta-analysis of 12 studies [24]. Other risky behaviors that might affect health have also been considered in the literature. For example, young drivers who self-report as more risk-loving, yet have a higher perceived likelihood of an accident, are more likely to adopt unsafe driving behaviors (i.e., speeding) [25].

Another related literature in agricultural economics has focused on decoupling risk perception and preferences to explain consumer purchasing and acceptance of food products given health risks [7–9, 12, 26]. Pennings et al. [7] evaluate the effects of risk perceptions and risk preferences on consumer responses to mad cow disease in three different countries. Both risk measures are collected using Likert scales and framed in the context of beef consumption. The authors find heterogeneous effects across countries where risk preferences are significant predictors for U.S. and German consumers’ beef consumption while risk perceptions are significant predictors for German and Dutch consumers. Lusk and Coble [8] find strong evidence that both risk perception and preferences significantly affect willingness to eat, purchase, and accept GM foods. However, risk perceptions have a relatively larger influence than risk preferences. This suggests that providing more information to consumers, especially in cases where risks may be overestimated, could significantly increase acceptance and purchases of GM foods. Focusing on beef consumption behaviors under risk of food-borne disease, Schroeder et al. [9] find the elasticity of consumer quantity reduction for risk perceptions to be approximately twice that of risk preferences among consumers in the United States, Canada, and Japan. Also, at the intersection of disasters and risk perceptions for food exposed to radiation, Ito and Kuriyama [27] argue that changing risk perceptions and information exposure may affect not only consumer willingness to pay (WTP) but also the distribution of WTP.

## Materials and methods

Our data were collected using a survey instrument administered online to a stratified sample of 972 adult residents of the United States during May 15–26, 2020 (the complete questionnaire can be found in the Supporting information). We dropped 36 respondents from the sample who have had a member of their household (including themselves) test positive for COVID-19. Results including these respondents are presented in S1 Appendix. Two additional respondents were removed from all analyses, one for reporting a 100-person household size, which we deemed an outlier, and another for not reporting household size. The final sample consisted of 934 valid responses. In addition to standard demographic data, the survey collected information about household characteristics related to COVID-19 such as income reductions due to the pandemic and presence of essential workers and immunocompromised and pregnant members in the household. Other questions in the survey elicited information about household behaviors and exposure to COVID-19.

The primary data collected and used in this analysis are also supplemented with secondary data that codify metropolitan and nonmetropolitan counties based on the 2013 rural-urban continuum code [28]. Pairing the state and county data collected from our respondents with the rural-urban continuum code allows each respondent to be assigned a corresponding classification code value. Under this classification system, the approximate population density of the county where the respondents reside is revealed. The coded values range from one to three for metro counties and four to nine for nonmetro counties. A higher code value indicates a more rural area. Indicator variables were generated for each classification code value with the most rural classification (i.e., a classification value of 9) used as the reference category. Including these categorical variables allows us to control for heterogeneities in risk preferences and perceptions across classifications and heterogeneities in the estimated correlation between these classifications and different risk-mitigation behaviors during COVID-19. This is particularly useful as COVID-19 is primarily spread via fine respiratory particulate matter and the spread is catalyzed by areas with a higher population that experienced elevated disease transmission and mortality compared to less populated areas [29].

Three risk measures were also collected in the survey. In the first measure, *self-assessed risk aversion* (SARA), respondents reported their general risk preferences on an 11-point Likert scale of increasing risk aversion with the endpoints labeled as ‘avoid all risk’ and ‘like to take risk’ and the midpoint indicating risk neutrality. The remaining two measures elicited risk perceptions and were contextualized under the COVID-19 disease. For one of those measures, we asked respondents to report their likelihood of exposure to COVID-19 delineated by a series of 14 different types of locations/activities: grocery store, hospital/clinic, doctor’s office, delivered packages, restaurant/bar, gas station, work, home, outdoor recreation, religious gathering, public transit (bus, plane, train), taxi/ride-share, daycare, and school. Respondents rated their likelihood of contracting COVID-19 at each of these locations/activities on a 5-point Likert scale. The scale ranges from ‘extremely unlikely’ to ‘extremely likely’ with a midpoint labeled as ‘neither likely nor unlikely’. Using the responses to each of the 14 locations/activities, we constructed the *number of risky places* (NRP) measure by counting the locations/activities where the subject rated the risk of contraction as either somewhat or extremely likely. As an alternative measure of risk perception associated with COVID-19, *perceived probability of contracting COVID-19* (PPCOV), we asked respondents to report the probability that someone in their household (including themselves) would contact COVID-19 in 2020. The scale used for this question ranged in integer increments from 0 to 100%.

## Results

### Descriptive statistics of sample

The respondents’ demographic and behavioral characteristics are summarized in Table 1. Approximately 48% of the subjects were male, and the average household size was between 2 and 3 members, with approximately 31% having children (under 18 years old) and 32% having elderly (65 years or older) as members of the household. The majority of respondents identified as either republican (32%) or democrat (40%). Approximately 38% reported a decrease in their income due to the COVID-19 pandemic.

**Table 1:** Summary of demographic and behavioral characteristics (n = 934).

Variable	%	Variable		%
Male	48	1 = Less than $10,000		6
Female	52	2 = $10,000–$14,999		5
		3 = $15,000–$24,999		9
1 = 18–24	11	4 = $25,000 –$34,999		11
2 = 25–34	17	5 = $35,000–$49,999		12
3 = 35–44	16	6 = $50,000–$74,999		18
4 = 45–54	17	7 = $75,000–$99,999		12
5 = 55–64	18	8 = $100,000–$149,999		14
6 = 65 or more	21	9 = $150,000–$199,999		7
		10 = $200,000 or more		6
Under 5-Years-Old	11			
6–12 Years Old	17	Income Decrease due to COVID-19		38
13–17 Years Old	14			
Under 18 (all categories)	31	Republican		32
		Democrat		40
65 Years or Older	32	Other		28
1 = Less than High School	2	Household Member Immunocompromised		19
2 = High School/GED	19	Household Member Pregnant		3
3 = Some College	23	Household Member Essential Worker		33
4 = Associates Degree	10			
5 = Bachelor’s Degree	30			
6 = Grad./Professional Degree	16			
		Mean	Median	Std. Dev.
Household Size Prior to COVID-19		2.64	2	1.44
Self-Assessed Risk Aversion (SARA)		6.04	6	2.55
Number of Risky Places (NRP)		7.53	9	4.09
Perceived Probability of Contracting COVID-19 (PPCOV)		31.71	30	24.66

*Notes*: Out of a sample of 970 respondents, 36 reported having a family member (including themselves) test positive for COVID-19 and were dropped from the analysis in this study.

For SARA, the average response on the 11-point scale was approximately 6, indicating slight risk averse behavior. With respect to NRP, of the 14 locations/activities in the list, subjects perceived exposure to COVID-19 as likely in an average of 7 locations/activities. The average PPCOV was approximately 33%.

Moving to other variables related to COVID-19, approximately 19% and 3% had a household member who was immunocompromised or pregnant, respectively, and 33% had a household member who was employed in an essential industry.

### Measures of risk perceptions

Looking more closely into the three different risk measures, we first examine the extent of the relationship between them by considering the correlation matrix in Table 2. Results from a Pearson correlation test indicate no significant correlation between SARA and NRP, a negative correlation between SARA and PPCOV, and a positive correlation between NRP and PPCOV. Although the correlations are relatively small in all cases, they indicate a lack of consistency between the risk preference and risk perception measures and warrant analysis of potential differences in the relationship between these measures and respondents’ decisions to follow risk-mitigation practices, which we consider in the next subsection.

**Table 2:** Correlation matrix between different risk measures (n = 934).

	Self-Assessed Risk Aversion (SARA)	Number of Risky Places (NRP)	Perceived Probability of Contracting COVID-19 (PPCOV)
Self-Assessed Risk Aversion (SARA)	1		
Number of Risky Places (NRP)	0.049	1	
	*p*-value = 0.137		
Perceived Probability of Contracting COVID-19 (PPCOV)	-0.098	0.239	1
	*p*-value = 0.003	*p*-value = 0.000	

**Fig 1:**
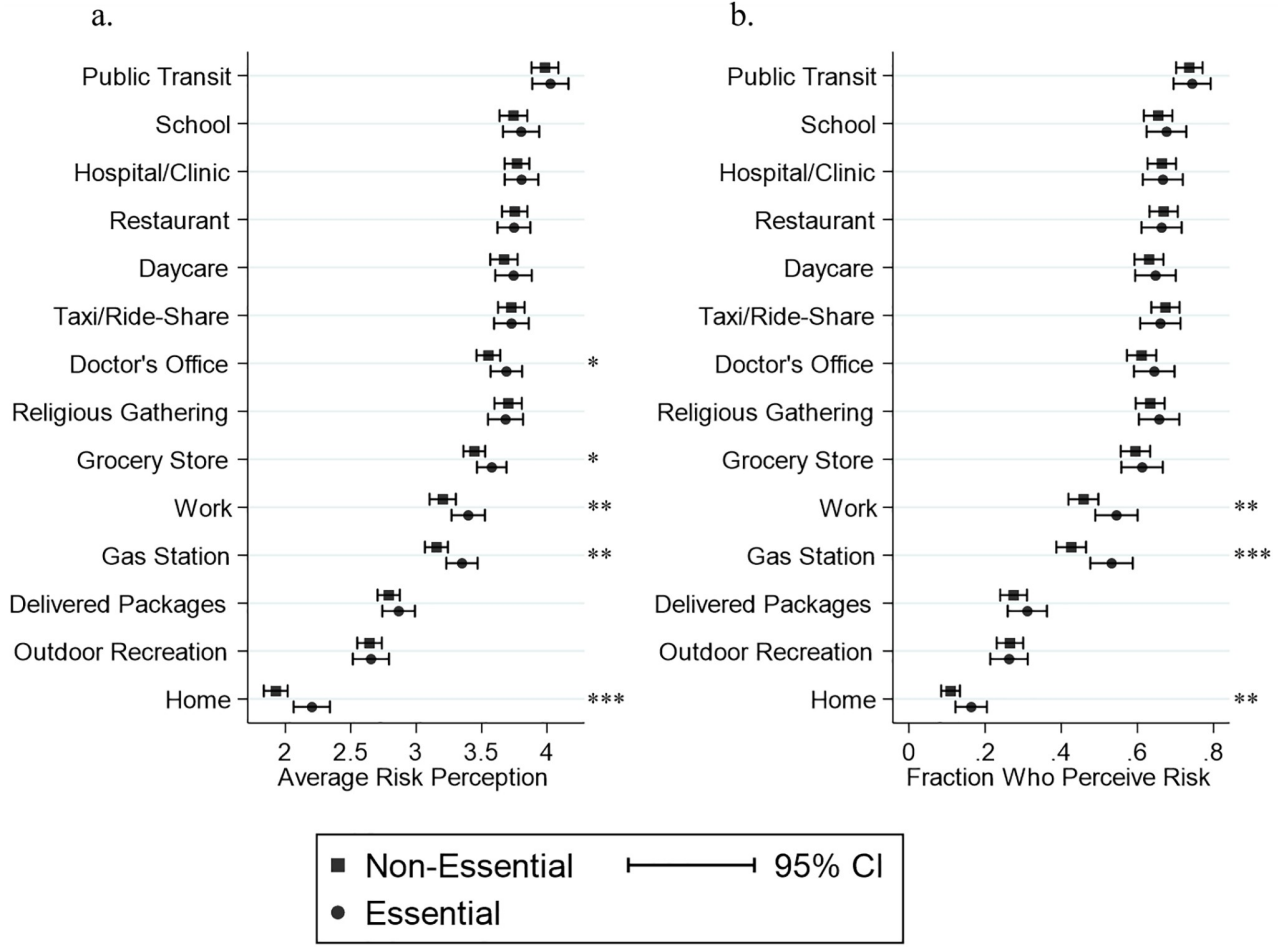
Perceived risk of infection in different places (essential vs. non-essential worker). For each variable, respondents indicated their perceived risk of contracting COVID-19 on a 5-point Likert scale (1 “extremely unlikely”; 2 “somewhat unlikely”; 3 “neither likely nor unlikely”; 4 “somewhat likely”; 5 “extremely likely”). The results are compared across respondents with vs. without an essential worker in the household. *, **, and *** indicate significant differences at the 90%, 95%, and 99% respectively. **(a)** The circles and squares indicate mean responses and the bars are 95% confidence intervals. **(b)** The circles and squares indicate the fraction of respondents who perceive a risk (i.e., responded “somewhat likely” or “extremely likely”) in each of the places/activities.

The respondents’ perception of the risk of exposure to COVID-19 in each of the 14 locations/activities is plotted in Fig 1. Average responses are shown in panel a, and the percentage of respondents who perceived the risk of exposure as “somewhat likely” or “extremely likely” (which we referred to as a positive risk) is shown in panel b. The results are broken down across the group of subjects who have a household member employed in an essential industry and those who do not. Respondents associated the highest risk of infection with public transit, and the lowest risk with staying at home. Additionally, more than half of the respondents associated a positive risk with schools, hospitals, restaurants, daycares, taxi/ride-shares, doctor’s offices, religious gatherings, and grocery stores. In fact, the perception of risk of infection in those places was very similar. As for the comparison of households with versus without an essential worker, it is interesting to note that the former had a significantly higher perception of risk associated with work, gas station, and home. Perhaps the additional exposure to COVID-19 from having a household member continue to work during the pandemic increased those respondents’ perception of the risk of the essential worker transmitting the virus in their homes.

**Fig 2:**
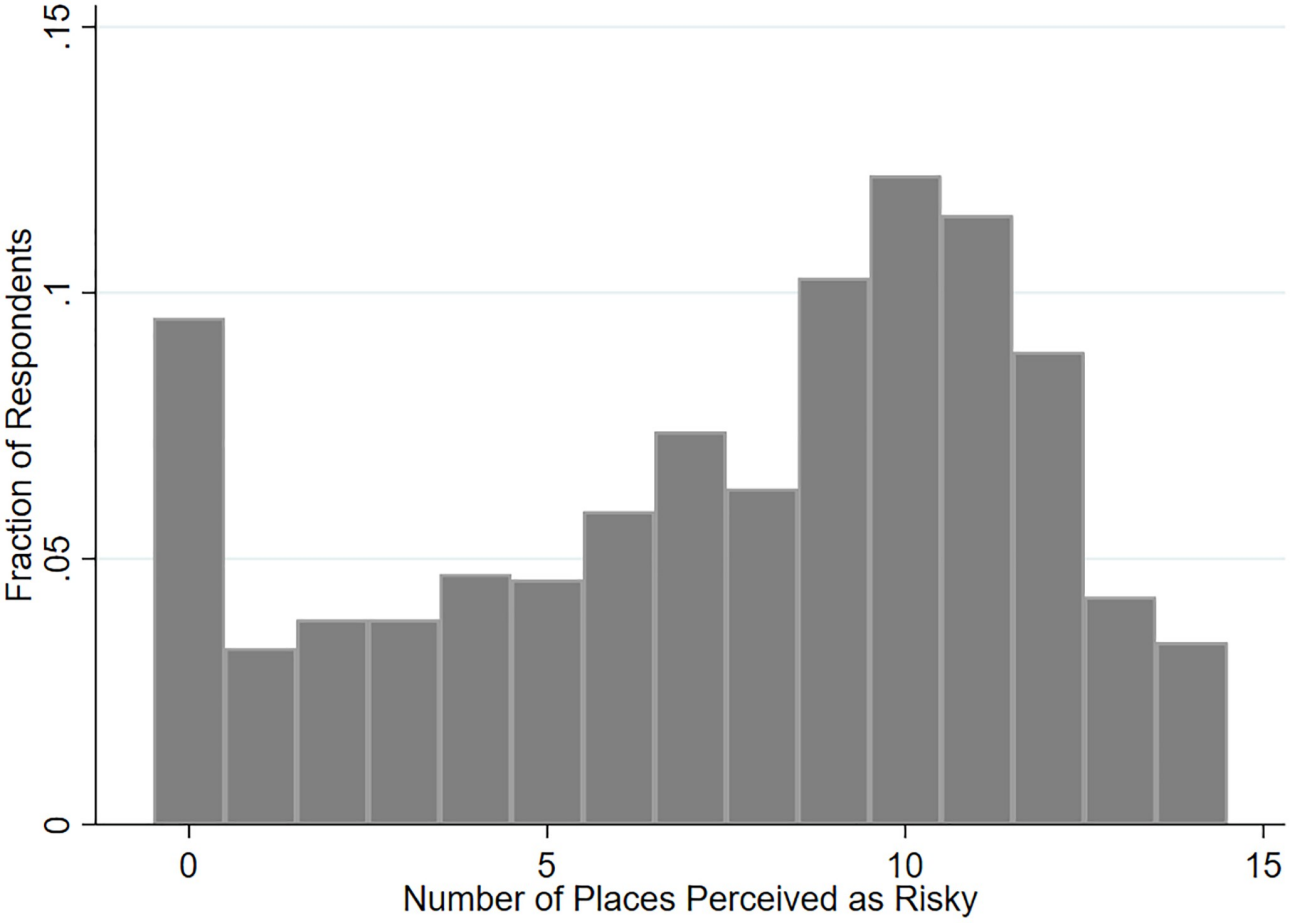
Histogram of the number of places people perceive as risky. The number of places respondents perceive as having positive risk (i.e., selected “somewhat risky” or “extremely risky”) was calculated. The fraction of individuals who perceive positive risk for different numbers of places is reported in the histogram.

Fig 2 presents a histogram of NRP. The modal response was 10 locations/activities, and we also observe a clustering around this point. An interesting point to note is the clustering at the endpoints of the histogram, where approximately 9% of respondents did not view any risk of infection, and another 4% perceived a positive risk with all 14 locations/activities listed. This indicates heterogeneity in opinions and risk perception toward COVID-19 and further motivates the analysis of the main individual and household characteristics that contribute to different perceptions and behaviors during the pandemic.

Regression analyses of the three risk measures examine the main factors influencing each measure, as shown in Table 3. Ordered Probit and Poisson regressions were estimated for the SARA and NRP measures, respectively, while a linear probability model was specified for PPCOV. To facilitate interpretation, odds ratios corresponding to the ordered Probit and Poisson regressions are presented in the first two columns in Table 3; a coefficient greater (less) than 1 indicates a positive (negative) relationship. Based on the results, respondents with immunocompromised or pregnant members in their household view more locations/activities as risky and have a higher perceived probability of contracting COVID-19. Household income was not significantly correlated with any of the risk measures, but respondents experiencing reductions in income due to COVID-19 reported significantly less risk-averse preferences. Considering individual characteristics, males were less risk averse and perceived lower probability of contracting COVID-19 compared to females, higher education decreased general risk aversion but increased the number of places perceived as risky, and republicans had a lower perceived probability of contracting COVID-19 and viewed fewer locations/activities as risky compared to democrats. As for other household characteristics, larger households were more perceptive of the risk of infection in more locations/activities, households with children under five years old perceived higher risk of contracting COVID-19, and households with elderly members reported more risk-averse preferences.

**Table 3:** Factors correlated with the three risk measures.

Variable	Self-Assessed Risk Aversion (SARA)	Number of Risky Places (NRP)	Perceived Probability of Contracting COVID-19 (PPCOV)
	Ordered Probit Odds Ratios	Poisson	LPM
		Odds Ratios	Coefficients
Immunocompromised/Pregnant Person in Household (1 if yes)	0.945	1.126***	5.319***
	(0.063)	(0.042)	(1.657)
Essential Worker in Household (1 if yes)	0.877*	1.018	1.110
	(0.067)	(0.038)	(1.930)
Household Income	0.981	0.996	0.039
	(0.015)	(0.009)	(0.311)
Reduced Income Due to COVID-19	0.866**	1.027	-0.305
	(0.051)	(0.045)	(1.820)
Male	0.727***	0.968	-3.039**
	(0.063)	(0.026)	(1.414)
Education	0.937**	1.034**	0.083
	(0.025)	(0.015)	(0.598)
Political Affiliation (Republican)	0.983	0.863***	-3.913**
	(0.071)	(0.041)	(1.842)
Political Affiliation (Other)	0.918	0.946	-3.301
	(0.068)	(0.036)	(2.303)
Household Size	0.981	1.045***	-1.176
	(0.037)	(0.016)	(0.922)
Children Under 5 (yes = 1)	0.803	1.041	5.833**
	(0.140)	(0.050)	(2.596)
Children Between 6 and 12 (yes = 1)	1.061	0.935	-2.715
	(0.106)	(0.059)	(2.784)
Children Between 13 and 17 (yes = 1)	0.919	1.050	3.838
	(0.122)	(0.047)	(2.481)
Elderly Present in Household (1 if yes)	1.290***	0.991	-2.100
	(0.108)	(0.039)	(2.244)
Rural-Urban Code Controls	Yes	Yes	Yes
R Squared/Log Likelihood	-2031.682	-2919.070	0.660

*Notes*: All models are estimated with state-level fixed effects using 934 observations. The first two columns report odds ratio estimates from an ordered Probit and Poisson regression, respectively. The third column reports coefficient estimates from a linear probability model (LPM). Standard errors in parentheses are clustered by state.

Significance levels:

*:10%

**:5%

***:1%.

### Analyzing risk-mitigation behavior

**Fig 3:**
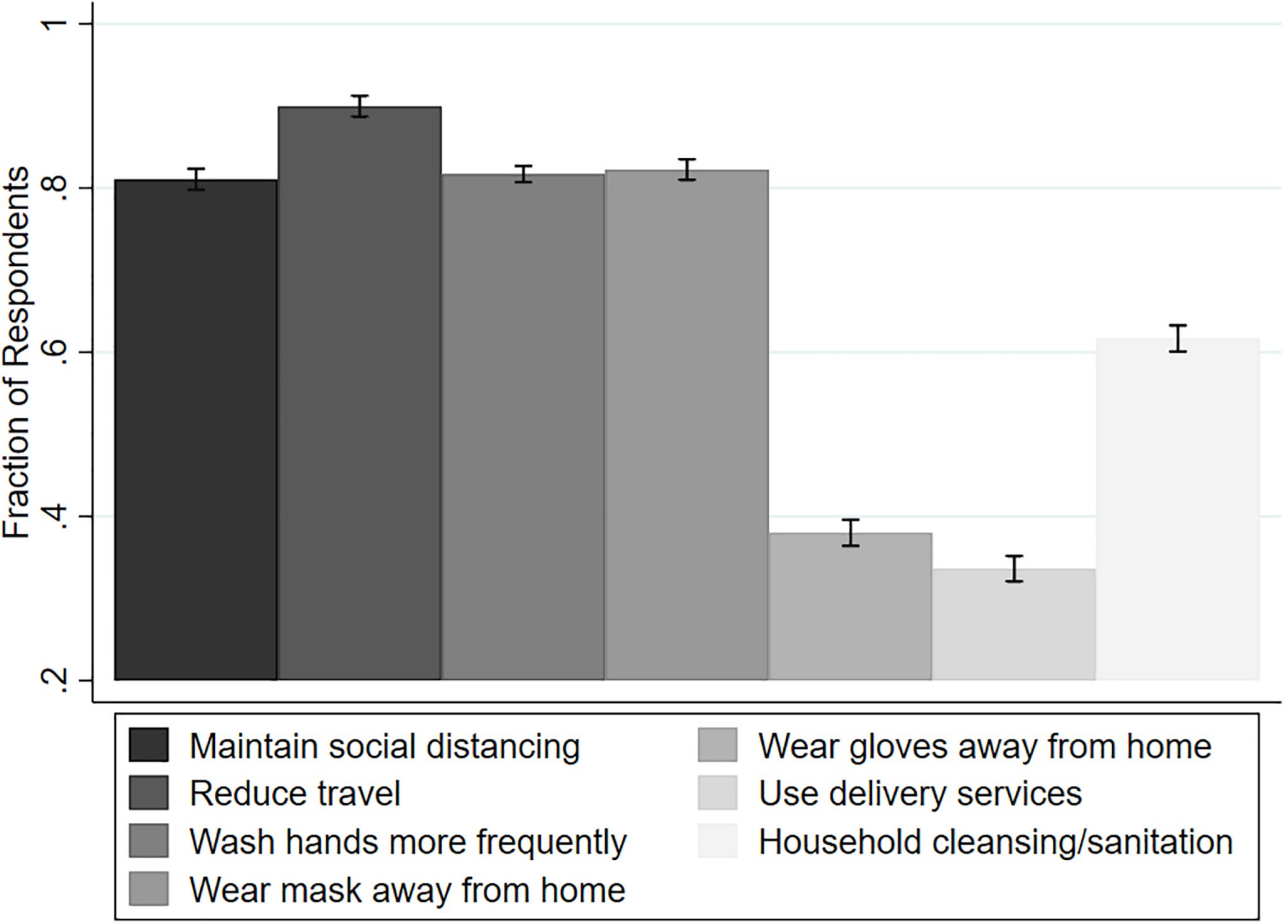
Fraction of respondents following each mitigation practice.

To understand how the three risk measures, as well as other factors, correlate with mitigation behaviors, we analyze the respondents’ adherence to various practices to protect against COVID-19 infections. The mitigation behaviors include maintaining social distancing, reducing travel, washing hands more frequently, wearing masks when away from home, wearing gloves when away from home, using delivery services, and performing additional household cleaning and sanitation. Fig 3 shows the fraction of respondents who reported following each practice. The most commonly adopted practices are social distancing, reduced travel, washing hands, and wearing a mask. We note a significantly higher tendency to wear a mask compared to gloves. In fact, more than 60% of subjects did not report wearing gloves as one of the mitigation practices during the pandemic. Another interesting point to note is the significantly greater attention toward personal hygiene compared to household hygiene, where the fraction practicing the former is almost 20 percentage points higher.

The respondents’ risk mitigation decisions are further analyzed with a system-of-equations Probit model using the seven mitigation practices as outcome variables. A system-of-equations model was specified to account for potential correlations in the error terms across the outcomes variables. We control for demographics, household characteristics, and state-level fixed effects. The odds ratios are reported in Table 4.

**Table 4:** Odds ratios for factors correlated with the likelihood of adopting mitigation bractices.

Variable	Social Distancing	Reduce Travel	Wash Hands	Wear Mask	Wear Gloves	Delivery Services	House Cleaning
SARA	1.058***	1.026	1.022	1.034***	1.049***	1.000	1.058***
	(0.023)	(0.023)	(0.029)	(0.014)	(0.017)	(0.013)	(0.021)
NRP	1.051***	1.048**	1.076***	1.042***	1.027**	1.023**	1.070***
	(0.014)	(0.020)	(0.015)	(0.011)	(0.014)	(0.011)	(0.010)
PPCOV	0.999	1.004*	0.999	0.999	1.001	1.003	0.998
	(0.002)	(0.002)	(0.002)	(0.002)	(0.002)	(0.002)	(0.002)
Immunocompr./ Pregnant	1.138	1.322**	0.953	0.997	0.984	1.197	1.297**
	(0.155)	(0.174)	(0.123)	(0.119)	(0.151)	(0.140)	(0.152)
Essential Worker in Household	0.975	0.972	0.805*	0.969	0.941	0.714***	0.807***
	(0.096)	(0.120)	(0.105)	(0.112)	(0.093)	(0.063)	(0.057)
Household Income	1.056***	1.069*	1.071**	1.038*	1.019	1.062**	1.038*
	(0.021)	(0.038)	(0.029)	(0.022)	(0.029)	(0.027)	(0.023)
Reduces Income Due to COVID-19	1.137	0.898	0.929	1.001	1.160	1.206	1.000
	(0.143)	(0.085)	(0.097)	(0.104)	(0.115)	(0.142)	(0.081)
Male	1.046	0.599***	0.893	0.850	1.171	1.176	0.854**
	(0.107)	(0.057)	(0.147)	(0.123)	(0.138)	(0.128)	(0.063)
Education	0.946	1.050	1.026	1.123**	1.102***	1.066**	0.995
	(0.036)	(0.059)	(0.043)	(0.051)	(0.041)	(0.034)	(0.036)
Political Affiliation (Republican)	0.754**	0.664***	1.206	0.748**	0.820	0.789**	1.099
	(0.096)	(0.087)	(0.202)	(0.087)	(0.117)	(0.077)	(0.113)
Political Affiliation (Other)	0.745***	1.037	0.811	0.951	0.881	0.871	0.927
	(0.083)	(0.141)	(0.105)	(0.127)	(0.082)	(0.091)	(0.110)
Household Size	0.832***	1.019	0.969	1.126*	1.054	0.994	1.011
	(0.032)	(0.057)	(0.036)	(0.068)	(0.044)	(0.048)	(0.046)
Children Under 5 (yes = 1)	1.012	0.538***	0.800	0.724*	1.169	1.571**	1.081
	(0.153)	(0.096)	(0.162)	(0.134)	(0.176)	(0.284)	(0.176)
Children Between 6 and 12 (yes = 1)	1.097	1.074	1.290	0.894	1.293*	1.716***	1.321*
	(0.152)	(0.192)	(0.231)	(0.139)	(0.177)	(0.336)	(0.222)
Children Between 13 and 17 (yes = 1)	1.168	0.827	1.113	0.974	1.240*	1.287	1.146
	(0.175)	(0.125)	(0.279)	(0.192)	(0.146)	(0.261)	(0.205)
Elderly Present in Household	1.322**	1.234**	1.579***	1.114	1.019	0.968	1.088
	(0.156)	(0.118)	(0.256)	(0.128)	(0.096)	(0.091)	(0.111)
Rural-Urban Code Controls	Yes	Yes	Yes	Yes	Yes	Yes	Yes

*Notes*: All models estimated using state-level fixed effects. A system of equations Probit model was estimated to account for potential correlation in the error terms across the outcomes variables. The coefficient estimate for the indicator variable on rural-urban continuum code = 8 is omitted from the third column (washing hands) as it does not vary across the dependent variable. The overall system-of-equations model estimated using 932 observations resulted in a log likelihood of -2786.113. Odds ratios are reported and standard errors are clustered by state and reported in parentheses.

Significance levels:

*:10%

**:5%

***:1%.

Examining the three risk measures, we observe a strong positive correlation between NRP and the likelihood of following all of the mitigation practices. This reasonably indicates that respondents who associate a risk of infection with a larger number of locations/activities are more likely to engage in each of the seven mitigation practices. SARA was also correlated with the respondents’ risk mitigation behavior, but the correlation was only statistically significant for some practices. Respondents who are more risk averse are more likely to maintain social distancing, wear a mask and gloves when away from home, and perform additional house cleaning and sanitation. Based on these results, it seems that domain-specific measures of risk perception (i.e., NRP) are a better predictor of mitigation behavior than domain-general measures of risk preferences (i.e., SARA). Surprisingly, however, we find no evidence that PPCOV is related to the likelihood of following any of the mitigation practices.

Having immunocompromised or pregnant members in the household increased the likelihood of reducing travel and performing additional household cleaning, whereas having a household member in an essential industry reduced the likelihood of using delivery services and performing additional household cleaning. Perhaps continuing to show up at work meant that essential workers had less time for additional household cleaning and more chance to pick up groceries and supplies from the store enroute to home rather than using a delivery service. Pre-COVID-19 income was positively correlated with the likelihood of maintaining social distancing, washing hands more often, and using delivery services, while income reductions due to COVID-19 were not correlated with the propensity to follow any of the mitigation practices.

The results also indicate that males were significantly less likely to reduce travel and perform additional household cleaning compared to females; education was positively correlated with wearing mask and gloves and using delivery services; and republicans were significantly less likely to maintain social distancing, reduce travel, wear a mask, and use delivery services compared to democrats. Household size was negatively correlated with maintaining social distancing. Having children in the household decreased the likelihood of reducing travel (for children under 5), but increased the likelihood of using delivery services (for children under 12 years), and having elderly in the household increased the likelihood of maintaining social distancing, reducing travel, and washing hands more frequently.

**Fig 4:**
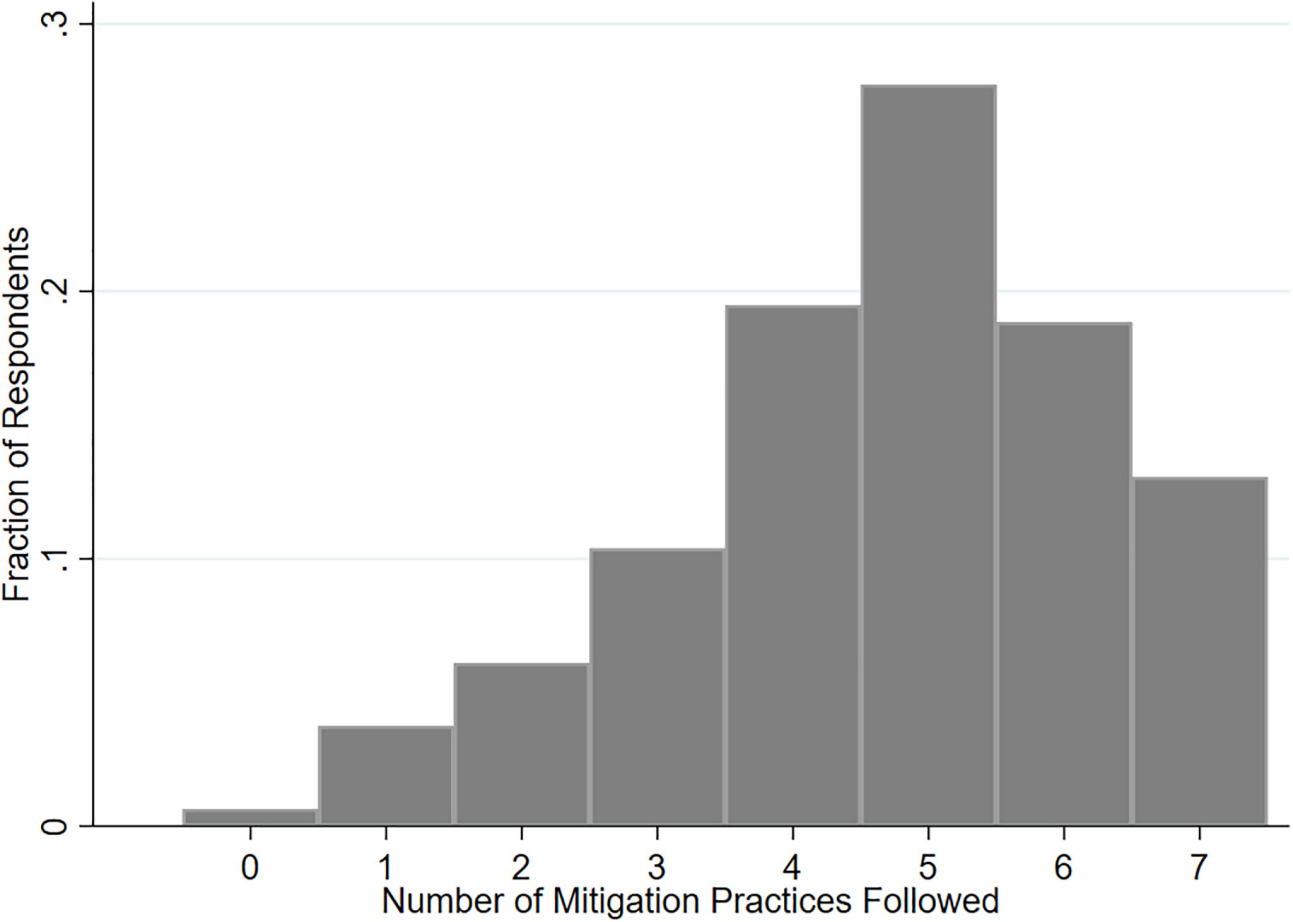
Histogram of the number of mitigation strategies practiced by different households. The number of mitigation practices followed by each respondent was calculated and the fraction who are following different numbers of practices is plotted in this histogram.

Considering the importance of following a combination of mitigation strategies, rather than just one, in preventing COVID-19 infections, we analyze the total number of practices followed by respondents and provide a histogram in Fig 4. The histogram seems to follow a left-skewed discrete normal distribution with a modal response at five practices. We also notice a significant fraction following four to six practices. This result indicates that a sizable portion of respondents are indeed adopting multiple mitigation practices to protect against COVID-19 infections. Notably, approximately 14% of respondents reported following all seven practices, and 10% reported following two or fewer practices. The fraction of respondents who are not following any practice is almost negligible (less than 1%).

Next, we estimate a Poisson regression to examine possible relationships between the respondents’ demographic and behavioral characteristics and the total number of mitigation practices they follow. The results are reported in Table 5 using the same covariates as Table 4. For ease of interpretation, the incidence rate ratios are reported so that a coefficient greater (less) than 1 indicates a positive (negative) relationship. The results in Table 5 conform to the previous analysis of individual mitigation practices. First, we observe that both SARA and NRP are significantly correlated with the total number of mitigation practices followed. This indicates that individuals who are more risk averse, and who perceive a risk of infection for more locations/activities, are more likely to adopt a higher number of mitigation practices to protect against COVID-19 infection. We also observe a slightly larger magnitude for the coefficient on the domain-specific measure of risk perceptions (i.e., NRP) compared to the domain-general measure of risk preferences (i.e., SARA), which is in line with the findings in Table 4. PPCOV again is not significantly correlated with respondents’ mitigation behavior. Looking at other variables in Table 5, we find that having a household member employed in an essential industry and identifying as a republican decreased the number of mitigation practices followed. Conversely, higher income, the presence of minor-aged children and elderly members in the household increased the number of mitigation practices followed.

**Table 5:** Odds ratios for factors correlated with the number of mitigation practices followed.

Variable	# of Mitigation Practices Followed
Self-Assessed Risk Aversion (SARA)	1.014***
	(0.003)
Number of Risky Places (NRP)	1.019***
	(0.003)
Perceived Probability of Contracting COVID-19 (PPCOV)	1.000
	(0.000)
Immunocompromised/Pregnant Person in Household	1.042*
	(0.023)
Essential Worker in Household	0.949***
	(0.017)
Household Income	1.019***
	(0.006)
Reduced Inc Due to COVID-19	1.026
	(0.023)
Male	0.983
	(0.023)
Education	1.015*
	(0.008)
Political Affiliation (Republican)	0.940***
	(0.018)
Political Affiliation (Other)	0.958
	(0.026)
Household Size	0.999
	(0.008)
Children Under 5 (yes = 1)	0.990
	(0.036)
Children Between 6 and 12 (yes = 1)	1.091***
	(0.030)
Children Between 13 and 17 (yes = 1)	1.047
	(0.037)
Elderly Present in Household	1.049**
	(0.021)
Rural-Urban Code Controls	Yes
Log Likelihood	-1795.759

*Notes*: A Poisson regression was estimated with state-level fixed effects using the number of mitigation strategies followed as the dependent variable with 934 observations. Incidence rate ratios are reported and standard errors are clustered by state and reported in parentheses.

Significance levels:

*:10%

**:5%

***:1%.

## Discussion and conclusion

The COVID-19 pandemic poses serious public health challenges, leading to significant lifestyle changes. Given the importance of adopting prevention strategies as an immediate response to the pandemic and potential means of slowing the spread of the virus, it is crucial to understand the main factors that influence individuals’ risk-mitigation behaviors. Our study takes a step forward in this direction by examining the extent to which risk perceptions and risk preferences correlate with individual decisions to adopt different mitigation practices. Building on previous literature in agricultural economics, we customize two measures of risk perceptions and one measure of risk preferences and investigate factors that influence these measures. In addition, we investigate differences in the way these risk measures correlate with the likelihood of following various mitigation practices and the total number of practices followed while controlling for other factors.

The results from this study add perspective to the existing literature on risk perceptions and preferences. While both SARA and NRP were significantly correlated with mitigation behaviors, NRP was correlated with a larger number of practices. This conforms to previous findings reported for consumption decisions of food products with potential health risks [7–9]; risk perceptions tend to influence behavior more than underlying risk preferences in decisions under risk. By showing the same pattern for mitigation behavior during a pandemic, our study demonstrates the robustness of this conclusion and extends it to another contextual setting. It is interesting to note, however, that unlike Seale et al. [5], we find that the perceived probability of contracting the disease (i.e., PPCOV) was not significantly correlated with individual risk-mitigation decisions. To this point, it is possible that risk perceptions in the context of COVID-19 are only being formed for specific locations/activities and that this construct impacts the perceived probability of contracting COVID-19. This being said, one limitation of this study, and other work in this vein, is that we are able to examine only associations between risk preferences, risk perceptions, and mitigation behaviors without making any causal claims. Here, we have modeled mitigation behavior as a function of both risk preferences and perceptions; however, it may be the case that risk perceptions are also a function of whether or not individuals adopt mitigation practices. In this sense, there may be a joint determination of risk perceptions and mitigation behaviors. One direction for future research may be to further unpack the causal mechanisms at work between risk perceptions and adoption behaviors.

The significant correlation between risk perceptions and mitigation behaviors found in this study provides useful implications for interventions promoting adherence to prevention strategies. As observed by Lusk and Coble [8], the malleable nature of risk perceptions, as opposed to the more stable risk preferences, makes them an ideal target for information-oriented strategies (e.g. audio, text, and visual messages) aimed at increasing people’s understanding of the virus and, therefore, nudging them toward adopting more virus mitigating behaviors. For example, educating individuals about the underlying risk of exposure to COVID-19 associated with different locations/activities may help change the public’s risk perceptions, which might, in turn, lead them to adopt a higher number of mitigation practices.

Another contribution of this study is highlighting heterogeneities in risk mitigation behavior based on individual and household characteristics, including gender, education level, political partisanship, household income, household size, and presence of children and elderly in the household. This information is useful in guiding policymakers to focus efforts on encouraging mitigation behaviors amongst groups where they are less common. Future research studies can also build on these findings by investigating the underlying factors leading to the observed heterogeneities and tailoring interventions to encourage mitigation behavior.

## Supporting information

S1 Appendix Survey questionnaire. (DOCX)
