# Supplementary material for: Perception versus preference: The role of self-assessed risk measures on individual mitigation behaviors during the COVID-19 pandemic
Source: PLoS One. 2021 Aug 4;16(8):e0254756. doi: 10.1371/journal.pone.0254756 (PMC8336792; doi:10.1371/journal.pone.0254756)
Supplement: S1 Appendix — (DOCX) [file pone.0254756.s001.docx]

**S1 Appendix. Survey Questionnaire**

Q2 What gender do you identify with?

- Male (1)
- Female (2)
- Other (3)

| 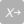 |
| --- |

Q3 How old are you?

- Under 18 (1)
- 18-24 (2)
- 25-34 (3)
- 35-44 (4)
- 45-54 (5)
- 55-64 (6)
- 65+ (7)

| Page Break |  |
| --- | --- |

Q4 Where do you currently reside?

State (1)

County (2)

▼ AL (1) ... WY ~ Weston (3192)

| 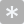 |
| --- |

Q5 What is your ZIP code?

________________________________________________________________

| Page Break |  |
| --- | --- |

| 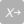 |
| --- |

Q6 What is the highest level of education you have completed? If a degree is in progress, please select the highest degree already achieved.

- Less than high school degree (1)
- High school graduate (high school diploma or equivalent including GED) (2)
- Some college but no degree (3)
- Associate degree in college (2-year) (4)
- Bachelor's degree in college (4-year) (5)
- Graduate or professional degree (6)

| 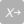 |
| --- |

Q7 Are you **currently** employed?

- Yes, full time (1)
- Yes, part time (2)
- No (3)

| Page Break |  |
| --- | --- |

| 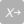 |
| --- |

Q8 What is your race/ethnicity? (please select all that apply)

- White (1)
- Black or African American (2)
- American Indian or Alaska Native (3)
- Asian (4)
- Native Hawaiian or Pacific Islander (5)
- Other (please specify) (6) ________________________________________________

| 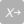 |
| --- |

Q9 Are you Spanish, Hispanic, or Latino?

- Yes (1)
- No (2)

| Page Break |  |
| --- | --- |

| 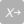 | 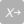 |
| --- | --- |

Q10 Are you currently...?

- Single/never married (1)
- Living together in a relationship (2)
- Married (3)
- Widowed (4)
- Divorced/separated (5)
- Other (please specify) (6) ________________________________________________

| Page Break |  |
| --- | --- |

| 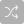 | 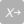 |
| --- | --- |

Q11 Generally speaking, do you usually think of yourself as a Republican, a Democrat, an Independent, or something else?

- Republican (1)
- Democrat (2)
- Independent (3)
- Other (please specify) (4) ________________________________________________
- No preference (5)

End of Block: Respondent Demographics

Start of Block: Household Demographics

| 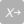 | 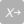 |
| --- | --- |

Q12 In 2019, which classification best describes your total household income in US dollars before taxes? (Please combine all incomes in your household)

- Under $10,000 (1)
- $10,000 to $14,999 (2)
- $15,000 to $24,999 (3)
- $25,000 to $34,999 (4)
- $35,000 to $49,999 (5)
- $50,000 to $74,999 (6)
- $75,000 to $99,999 (7)
- $100,000 to $149,999 (8)
- $150,000 to $199,999 (9)
- $200,000 or more (10)

| Page Break |  |
| --- | --- |

| 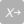 |
| --- |

Q13 Are any members of your household employed in essential industries where they are required to report to work during the COVID-19 pandemic?

- Yes (1)
- No (2)

| Page Break |  |
| --- | --- |

Display This Question:

If Are any members of your household employed in essential industries where they are required to rep... = Yes

Q14 What essential industries are the members of your household working in?

________________________________________________________________

| Page Break |  |
| --- | --- |

| 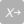 |
| --- |

Q15 Are any members of your household employed in the healthcare industry?

- Yes (1)
- No (2)

| Page Break |  |
| --- | --- |

| 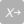 |
| --- |

Q16 Are any members of your household immunocompromised?

- Yes (1)
- No (2)

| 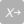 |
| --- |

Q17 Are any members of your household pregnant?

- Yes (1)
- No (2)

End of Block: Household Demographics

Start of Block: Household COVID-19 Behaviors and Exposure

Q18 The following questions explore how your household has been affected by and responded to the COVID-19 pandemic.

| 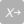 |
| --- |

Q19 Before the COVID-19 pandemic, please indicate how many individuals were residing in your household for each of the following categories **including yourself.**

 Before COVID-19 if your household had no members in a particular age range please enter zero.

|  | Children aged 2 or younger (1) | Children aged 3-5 (2) | Children aged 6-12 (3) | Children aged 13-17 (4) | Adults aged 18-64 (5) | Adults aged 65 or over (6) |
| --- | --- | --- | --- | --- | --- | --- |
| Number of Individuals (1) |  |  |  |  |  |  |

| Page Break |  |
| --- | --- |

| 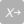 |
| --- |

Q20 During the COVID-19 pandemic, please indicate the number of individuals residing in your household for each of the following categories **including yourself.**


During COVID-19 if your household had no members in a particular age range please enter zero.

|  | Children aged 2 or younger (1) | Children aged 3-5 (2) | Children aged 6-12 (3) | Children aged 13-17 (4) | Adults aged 18-64 (5) | Adults aged 65 or over (6) |
| --- | --- | --- | --- | --- | --- | --- |
| Number of Individuals (1) |  |  |  |  |  |  |

| Page Break |  |
| --- | --- |

| 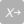 |
| --- |

Q21 Has your **household income** changed due to the COVID-19 pandemic?

- Decreased (1)
- No change (2)
- Increased (3)

| Page Break |  |
| --- | --- |

Display This Question:

If Has your household income changed due to the COVID-19 pandemic? = Decreased

Or Has your household income changed due to the COVID-19 pandemic? = Increased

Q22 By what percentage did your household income change due to the COVID-19 pandemic?

|  | 0 | 10 | 20 | 30 | 40 | 50 | 60 | 70 | 80 | 90 | 100 |
| --- | --- | --- | --- | --- | --- | --- | --- | --- | --- | --- | --- |

| Please drag the slider to select the percentage change in household income () | 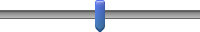 |
| --- | --- |

Display This Question:

If Has your household income changed due to the COVID-19 pandemic? = Decreased

| 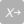 |
| --- |

Q23 In what ways was your total household income reduced due to the COVID-19 pandemic? Please select all that apply:

- Reduced hours (1)
- Layoff/furlough (2)
- Unpaid time off for sick leave (3)
- Reduced pay (4)
- Employer went out of business (5)
- Self-employed went out of business (6)
- Loss of commission/bonus (7)
- Other (8)

| Page Break |  |
| --- | --- |

Display This Question:

If In what ways was your total household income reduced due to the COVID-19 pandemic? Please select... = Other

Q24 What other ways was your total household income reduced due to the COVID-19 pandemic?

________________________________________________________________

________________________________________________________________

________________________________________________________________

________________________________________________________________

________________________________________________________________

| Page Break |  |
| --- | --- |

| 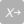 |
| --- |

Q25 Which of the following measures is your household taking to limit the risk of contracting COVID-19?

- Maintaining 6 feet of space between people (1)
- Reducing non-essential trips away from home (2)
- Washing hands more frequently (3)
- Wearing a mask when away from home (4)
- Wearing gloves when away from home (5)
- Using delivery services (e.g. grocery pickup) (6)
- Additional household cleaning/sanitation (7)
- Other (8)

| Page Break |  |
| --- | --- |

Display This Question:

If Which of the following measures is your household taking to limit the risk of contracting COVID-19? = Other

Q26 What other measures is your household taking to limit the risk of contracting COVID-19?

________________________________________________________________

________________________________________________________________

________________________________________________________________

________________________________________________________________

________________________________________________________________

| Page Break |  |
| --- | --- |

| 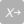 |
| --- |

Q27 How long are you willing to maintain these household measures to reduce the risk of contracting COVID-19? Please select the maximum amount of time from the dropdown list below:

▼ 1 month (1) ... More than 12 months (13)

| Page Break |  |
| --- | --- |

| 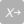 |
| --- |

Q28 Have you or any household members self-isolated due to concerns about exposure to COVID-19?

- Yes (1)
- No (2)

| 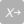 |
| --- |

Q29 Have you or any household members tested positive for COVID-19?

- Yes (1)
- No (2)

Display This Question:

If Have you or any household members tested positive for COVID-19? = Yes

| 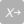 |
| --- |

Q30 Have you or any household members been hospitalized for COVID-19?

- Yes (1)
- No (2)

| Page Break |  |
| --- | --- |

Q31 How likely do you think it is that you or a family member will contract COVID-19 in 2020?

|  | 0 | 10 | 20 | 30 | 40 | 50 | 60 | 70 | 80 | 90 | 100 |
| --- | --- | --- | --- | --- | --- | --- | --- | --- | --- | --- | --- |

| Please drag the slider to select the percent chance () | 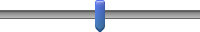 |
| --- | --- |

| Page Break |  |
| --- | --- |

| 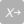 |
| --- |

Q32 Please select the dates you think the following events will occur:

|  | Month | Year |
| --- | --- | --- |
|  |  |  |
| How soon do you believe the COVID-19 pandemic will end? (1) | ▼ January (1 ... December (12) | ▼ 2020 (1 ... 2030 (11) |
| When do you expect the economy will return to normal? (2) | ▼ January (1 ... December (12) | ▼ 2020 (1 ... 2030 (11) |

End of Block: Household COVID-19 Behaviors and Exposure

Start of Block: ERP Status

Q33 In recent weeks, the United States Congress passed a one-time Economic Impact Payment for individuals affected by the COVID-19 pandemic.


According to the U.S. Internal Revenue Service: Tax filers with adjusted gross income up to $75,000 for individuals and up to $150,000 for married couples filing joint returns will receive the full payment. 
For filers with income above those amounts, the payment amount is reduced by $5 for each $100 above the $75,000/$150,000 thresholds. Single filers with income exceeding $99,000 and $198,000 for joint filers with no children are not eligible. Social Security recipients and railroad retirees who are otherwise not required to file a tax return are also eligible and will not be required to file a return. 
Eligible taxpayers who filed tax returns for either 2019 or 2018 will automatically receive an economic impact payment of up to $1,200 for individuals or $2,400 for married couples and up to $500 for each qualifying child.

| 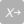 |
| --- |

Q34 Have you already received an Economic Impact Payment?

- Yes (1)
- No (2)

| Page Break |  |
| --- | --- |

Display This Question:

If Have you already received an Economic Impact Payment? = No

| 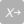 |
| --- |

Q35 Based on the description above, do you believe your household will receive an Economic Impact Payment from this program?

- Yes (1)
- No (2)

End of Block: ERP Status

Start of Block: ERP Spending Questions - Anticipate but have not yet received

| 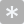 |
| --- |

Q36 How much do you think your household will receive? Please enter the total dollar amount below:

________________________________________________________________

| Page Break |  |
| --- | --- |

| 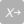 |
| --- |

Q37 Suppose you receive the full ${Q36/ChoiceTextEntryValue} dollars you anticipate from the Economic Impact Payment. In which of the following categories will you use the money? Please select all that apply.

- Food (1)
- Shelter (e.g. rent, mortgage, utilities) (2)
- Home improvement/repairs/maintenance (3)
- Durable goods (e.g. appliances, electronics, furniture) (4)
- Leisure or entertainment (5)
- Savings or investment (6)
- Charitable donation (7)
- Childcare (8)
- Travel (9)
- Other (10)

| Page Break |  |
| --- | --- |

Carry Forward Selected Choices from "Suppose you receive the full ${q://QID59/ChoiceTextEntryValue} dollars you anticipate from the Economic Impact Payment. In which of the following categories will you use the money? Please select all that apply. "

| 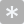 | 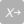 |
| --- | --- |

Q38 Think about what you would spend on each of the previously selected categories below. What percentage of the ${Q36/ChoiceTextEntryValue} dollar Economic Impact Payment will you allocate to each of the following areas?

Food : _______ (1)

Shelter (e.g. rent, mortgage, utilities) : _______ (2)

Home improvement/repairs/maintenance : _______ (3)

Durable goods (e.g. appliances, electronics, furniture) : _______ (4)

Leisure or entertainment : _______ (5)

Savings or investment : _______ (6)

Charitable donation : _______ (7)

Childcare : _______ (8)

Travel : _______ (9)

Other : _______ (10)

Total : ________

| Page Break |  |
| --- | --- |

Display This Question:

If Suppose you receive the full ${q://QID59/ChoiceTextEntryValue} dollars you anticipate from the Ec... = Other

Q39 What other things will you use the money from the Economic Impact Payment to purchase? Please list all that apply:

________________________________________________________________

________________________________________________________________

________________________________________________________________

________________________________________________________________

________________________________________________________________

| Page Break |  |
| --- | --- |

Display This Question:

If Suppose you receive the full ${q://QID59/ChoiceTextEntryValue} dollars you anticipate from the Ec... = Food

| 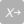 |
| --- |

Q40 Of the ${Q38/ChoiceNumericEntryValue/x1} percent of the impact payment you would spend on food, which of the following would you purchase? Please select all that apply:

- Groceries from a supermarket to prepare at home (1)
- Pre-prepared foods to eat at home (e.g. deli, bakery, etc.) (2)
- Delivery/takeout from a chain restaurant (3)
- Delivery/takeout from a locally-owned restaurant (4)
- Locally produced food (e.g. farmers market, CSA, etc.) (5)

| Page Break |  |
| --- | --- |

Display This Question:

If Suppose you receive the full ${q://QID59/ChoiceTextEntryValue} dollars you anticipate from the Ec... = Charitable donation

| 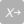 |
| --- |

Q41 Of the ${Q38/ChoiceNumericEntryValue/x11} percent of the impact payment you would allocate to charitable donations, where would you give the money? Please select all that apply:

- Religious organization (1)
- Hospital or medical organization (2)
- Family members or friends in need (3)
- Other (please specify): (4) ________________________________________________

| Page Break |  |
| --- | --- |

Display This Question:

If Suppose you receive the full ${q://QID59/ChoiceTextEntryValue} dollars you anticipate from the Ec... = Leisure or entertainment

| 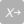 |
| --- |

Q42 Of the ${Q38/ChoiceNumericEntryValue/x4} percent of the impact payment you would allocate to leisure and entertainment spending, which of the following would you purchase? Please select all that apply:

- Streaming Services (Netflix/Hulu/Disney+) (1)
- Outdoor activities (sports, hiking, water-sports) (2)
- Exercise equipment (3)
- Board games, video games, computer (4)
- Other (please specify): (5) ________________________________________________

| Page Break |  |
| --- | --- |

| 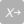 |
| --- |

Q43 How quickly do you expect to spend your Economic Impact Payment once you receive it?

- within 1 week (1)
- 1-2 weeks (2)
- 2-3 weeks (3)
- 3-4 weeks (4)
- over 4 weeks (5)

End of Block: ERP Spending Questions - Anticipate but have not yet received

Start of Block: ERP Spending Questions - Already received

| 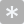 |
| --- |

Q44 How much of an Economic Impact Payment did your household receive? Please enter the total dollar amount below:

________________________________________________________________

| Page Break |  |
| --- | --- |

| 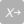 |
| --- |

Q45 In which of the following categories will you use the ${Q44/ChoiceTextEntryValue} dollar Economic Impact Payment? Please select all that apply.

- Food (1)
- Shelter (e.g. rent, mortgage, utilities) (2)
- Home improvement/repairs/maintenance (3)
- Durable goods (e.g. appliances, electronics, furniture) (4)
- Leisure or entertainment (5)
- Savings or investment (6)
- Charitable donation (7)
- Childcare (8)
- Travel (9)
- Other (10)

| Page Break |  |
| --- | --- |

Carry Forward Selected Choices from "In which of the following categories will you use the ${q://QID161/ChoiceTextEntryValue} dollar Economic Impact Payment? Please select all that apply. "

| 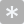 | 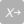 |
| --- | --- |

Q46 Think about what you would spend on each of the previously selected categories below. What percentage of the ${Q44/ChoiceTextEntryValue} dollar Economic Impact Payment will you allocate to each of the following areas?

Food : _______ (1)

Shelter (e.g. rent, mortgage, utilities) : _______ (2)

Home improvement/repairs/maintenance : _______ (3)

Durable goods (e.g. appliances, electronics, furniture) : _______ (4)

Leisure or entertainment : _______ (5)

Savings or investment : _______ (6)

Charitable donation : _______ (7)

Childcare : _______ (8)

Travel : _______ (9)

Other : _______ (10)

Total : ________

| Page Break |  |
| --- | --- |

Display This Question:

If In which of the following categories will you use the ${q://QID161/ChoiceTextEntryValue} dollar E... = Other

Q47 What other things will you use the money from the Economic Impact Payment to purchase? Please list all that apply:

________________________________________________________________

________________________________________________________________

________________________________________________________________

________________________________________________________________

________________________________________________________________

| Page Break |  |
| --- | --- |

Display This Question:

If In which of the following categories will you use the ${q://QID161/ChoiceTextEntryValue} dollar E... = Food

| 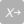 |
| --- |

Q48 Of the ${Q46/ChoiceNumericEntryValue/x1} percent of the impact payment you would spend on food, which of the following would you purchase? Please select all that apply:

- Groceries from a supermarket to prepare at home (1)
- Pre-prepared foods to eat at home (e.g. deli, bakery, etc.) (2)
- Delivery/takeout from a chain restaurant (3)
- Delivery/takeout from a locally-owned restaurant (4)
- Locally produced food (e.g. farmers market, CSA, etc.) (5)

| Page Break |  |
| --- | --- |

Display This Question:

If In which of the following categories will you use the ${q://QID161/ChoiceTextEntryValue} dollar E... = Charitable donation

| 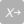 |
| --- |

Q49 Of the ${Q46/ChoiceNumericEntryValue/x11} percent of the impact payment you would allocate to charitable donations, where would you give the money? Please select all that apply:

- Religious organization (1)
- Hospital or medical organization (2)
- Family members or friends in need (3)
- Other (please specify): (4) ________________________________________________

| Page Break |  |
| --- | --- |

Display This Question:

If In which of the following categories will you use the ${q://QID161/ChoiceTextEntryValue} dollar E... = Leisure or entertainment

| 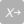 |
| --- |

Q50 Of the ${Q46/ChoiceNumericEntryValue/x4} percent of the impact payment you would allocate to leisure and entertainment spending, which of the following would you purchase? Please select all that apply:

- Streaming Services (Netflix/Hulu/Disney+) (1)
- Outdoor activities (sports, hiking, water-sports) (2)
- Exercise equipment (3)
- Board games, video games, computer (4)
- Other (please specify): (5) ________________________________________________

| Page Break |  |
| --- | --- |

| 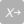 |
| --- |

Q51 How quickly do you expect to spend your Economic Impact Payment?

- within 1 week (1)
- 1-2 weeks (2)
- 2-3 weeks (3)
- 3-4 weeks (4)
- over 4 weeks (5)

End of Block: ERP Spending Questions - Already received

Start of Block: ERP Spending Questions - Do not anticipate

Q52 For the following questions, suppose the government introduces another Economic Impact Payment where your household receives a one-time payment of $1000.

| 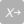 |
| --- |

Q53 Suppose you receive a $1000 Economic Impact Payment. In which of the following categories would you use the money? Please select all that apply.

- Food (1)
- Shelter (e.g. rent, mortgage, utilities) (2)
- Home improvement/repairs/maintenance (3)
- Durable goods (e.g. appliances, electronics, furniture) (4)
- Leisure or entertainment (5)
- Savings or investment (6)
- Charitable donation (7)
- Childcare (8)
- Travel (9)
- Other (10)

| Page Break |  |
| --- | --- |

Carry Forward Selected Choices from "Suppose you receive a $1000 Economic Impact Payment. In which of the following categories would you use the money? Please select all that apply. "

| 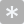 | 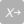 |
| --- | --- |

Q54 Think about what you would spend on each of the previously selected categories below. What percentage of the $1000 Economic Impact Payment would you allocate to each of the following areas?

Food : _______ (1)

Shelter (e.g. rent, mortgage, utilities) : _______ (2)

Home improvement/repairs/maintenance : _______ (3)

Durable goods (e.g. appliances, electronics, furniture) : _______ (4)

Leisure or entertainment : _______ (5)

Savings or investment : _______ (6)

Charitable donation : _______ (7)

Childcare : _______ (8)

Travel : _______ (9)

Other : _______ (10)

Total : ________

| Page Break |  |
| --- | --- |

Display This Question:

If Suppose you receive a $1000 Economic Impact Payment. In which of the following categories would y... = Other

Q55 What other things would you use the money from the Economic Impact Payment to purchase? Please list all that apply:

________________________________________________________________

________________________________________________________________

________________________________________________________________

________________________________________________________________

________________________________________________________________

| Page Break |  |
| --- | --- |

Display This Question:

If Suppose you receive a $1000 Economic Impact Payment. In which of the following categories would y... = Food

| 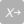 |
| --- |

Q56 Of the ${Q54/ChoiceNumericEntryValue/x1} percent of the impact payment you would spend on food, which of the following would you purchase? Please select all that apply:

- Groceries from a supermarket to prepare at home (1)
- Pre-prepared foods to eat at home (e.g. deli, bakery, etc.) (2)
- Delivery/takeout from a chain restaurant (3)
- Delivery/takeout from a locally-owned restaurant (4)
- Locally produced food (e.g. farmers market, CSA, etc.) (5)

| Page Break |  |
| --- | --- |

Display This Question:

If Suppose you receive a $1000 Economic Impact Payment. In which of the following categories would y... = Charitable donation

| 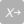 |
| --- |

Q57 Of the ${Q54/ChoiceNumericEntryValue/x11} percent of the impact payment you would allocate to charitable donations, where would you give the money? Please select all that apply:

- Religious organization (1)
- Hospital or medical organization (2)
- Family members or friends in need (3)
- Other (please specify): (4) ________________________________________________

| Page Break |  |
| --- | --- |

Display This Question:

If Suppose you receive a $1000 Economic Impact Payment. In which of the following categories would y... = Leisure or entertainment

| 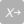 |
| --- |

Q58 Of the ${Q54/ChoiceNumericEntryValue/x4} percent of the impact payment you would allocate to leisure and entertainment spending, which of the following would you purchase? Please select all that apply:

- Streaming Services (Netflix/Hulu/Disney+) (1)
- Outdoor activities (sports, hiking, water-sports) (2)
- Exercise equipment (3)
- Board games, video games, computer (4)
- Other (please specify): (5) ________________________________________________

| Page Break |  |
| --- | --- |

| 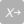 |
| --- |

Q59 How quickly would you expect to spend your Economic Impact Payment after receiving it?

- within 1 week (1)
- 1-2 weeks (2)
- 2-3 weeks (3)
- 3-4 weeks (4)
- over 4 weeks (5)

End of Block: ERP Spending Questions - Do not anticipate

Start of Block: Changing grocery expenditures due to COVID-19

| 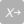 |
| --- |

Q60 Who in the household is taking trips to the store during the COVID-19 pandemic?

- Myself (1)
- My partner/spouse and I (2)
- Only my partner/spouse (3)
- Other (please specify): (4) ________________________________________________

| 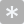 |
| --- |

Q61 How many trips to the grocery store has your household made in the last 30 days?

________________________________________________________________

| Page Break |  |
| --- | --- |

| 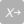 |
| --- |

Q62 Since the COVID-19 pandemic started, have any of the following items been unavailable to you while grocery shopping? Select all that apply.

- Produce (1)
- Meat (2)
- Dry goods (e.g. pasta, rice, beans, etc.) (3)
- Paper products (e.g. toilet paper, paper towels, tissues, etc.) (4)
- Beauty products (5)
- Medical/pharmacy supplies (6)
- Water, flavored drinks, and other non-alcoholic beverages (7)
- Alcoholic beverages (8)
- Other (please specify): (9) ________________________________________________

| Page Break |  |
| --- | --- |

| 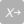 |
| --- |

Q63 Are you more likely to plan a shopping list of things to purchase during the COVID-19 pandemic?

- Yes (1)
- No (2)

| Page Break |  |
| --- | --- |

Q64 For the following goods, please indicate how the quantities you are purchasing has changed during the COVID-19 pandemic compared to before COVID-19.

|  | Decreased A Lot (44) | Decreased A Little (45) | No Change (46) | Increased A Little (47) | Increased A Lot (48) |
| --- | --- | --- | --- | --- | --- |
| Fresh fruits/vegetables (1) |  |  |  |  |  |
| Frozen fruits/vegetables (2) |  |  |  |  |  |
| Canned/non-perishable food items (13) |  |  |  |  |  |
| Dry goods (rice, cereal, beans, pasta) (14) |  |  |  |  |  |
| Snack food items (chips, cookies) (15) |  |  |  |  |  |
| Store prepared foods (20) |  |  |  |  |  |
| Frozen prepared meals (pizza, etc.) (16) |  |  |  |  |  |
| Dairy (e.g. cheese, eggs, milk, etc.) (3) |  |  |  |  |  |
| Meat (e.g. chicken, beef, pork, etc.) (11) |  |  |  |  |  |
| Wine, beer, liquor (17) |  |  |  |  |  |
| Please select decreased a little for this row. Thank you for reading carefully. (21) |  |  |  |  |  |

| Page Break |  |
| --- | --- |

| 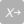 |
| --- |

Q65
Are you spending more or less at the following locations during the COVID-19 pandemic compared to before COVID-19?

|  | N/A (I don't shop here) (1) | A Lot Less (2) | A Little Less (3) | About the same (4) | A Little More (5) | A Lot More (6) |
| --- | --- | --- | --- | --- | --- | --- |
| Grocery stores (7) |  |  |  |  |  |  |
| Fast-food outlets (1) |  |  |  |  |  |  |
| Convenience Stores (8) |  |  |  |  |  |  |
| Delivery (5) |  |  |  |  |  |  |
| Takeout/pickup/carryout (6) |  |  |  |  |  |  |

| Page Break |  |
| --- | --- |

| 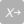 |
| --- |

Q66 During COVID-19, has your household used a local food pantry to help meet any household needs?

- Yes (1)
- No (2)

| Page Break |  |
| --- | --- |

| 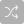 | 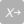 |
| --- | --- |

Q67 Please rank the following food characteristics in order of their importance to you when shopping during the COVID-19 pandemic. Please drag and drop your choices to rank them. A ranking of 1 is the most important characteristic and a ranking of 10 is the least important characteristic.

______ Non-perishable/shelf stable (1)

______ Fresh (2)

______ Cost (3)

______ Nutrition (4)

______ Untouched goods on the back of the shelf (5)

______ Local (6)

______ Organic (7)

______ Sustainably produced (8)

______ Convenience (9)

______ Brand (10)

| Page Break |  |
| --- | --- |

| 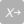 |
| --- |

Q68 Do you think your overall diet and nutrition has improved during the COVID-19 pandemic?

- Yes (1)
- No (2)

| Page Break |  |
| --- | --- |

| 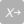 |
| --- |

Q69 How likely are you to be exposed to COVID-19 at each of the following locations:

|  | Extremely unlikely (1) | Somewhat unlikely (2) | Neither likely nor unlikely (3) | Somewhat likely (4) | Extremely likely (5) |
| --- | --- | --- | --- | --- | --- |
| Grocery Store (1) |  |  |  |  |  |
| Hospital/Clinic (4) |  |  |  |  |  |
| Doctor's Office (2) |  |  |  |  |  |
| Delivered Packages (5) |  |  |  |  |  |
| Restaurant/Bar (10) |  |  |  |  |  |
| Gas Station (6) |  |  |  |  |  |
| Work (7) |  |  |  |  |  |
| Home (8) |  |  |  |  |  |
| Outdoor Recreation (9) |  |  |  |  |  |
| Religious Gathering (11) |  |  |  |  |  |
| Public Transit (Bus, Plane, Train) (13) |  |  |  |  |  |
| Taxi/Ride-share (15) |  |  |  |  |  |
| Daycare (17) |  |  |  |  |  |
| School (16) |  |  |  |  |  |

End of Block: Changing grocery expenditures due to COVID-19

Start of Block: Risk and personality questions

Q70 On the scale below, do you view yourself as a someone who avoids risks or someone who is a risk taker?

|  | Avoid all risks | Neutral about risk | Likes to take risks |
| --- | --- | --- | --- |

|  | 0 | 1 | 2 | 3 | 4 | 5 | 6 | 7 | 8 | 9 | 10 |
| --- | --- | --- | --- | --- | --- | --- | --- | --- | --- | --- | --- |

| Please drag the slider to select the number corresponding to your risk preferences. () | 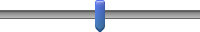 |
| --- | --- |

| 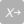 |
| --- |

Q71 Please indicate how well each of the following describes you?

|  | A lot (1) | Some (2) | A little (3) | Not at all (4) |
| --- | --- | --- | --- | --- |
| Outgoing (1) |  |  |  |  |
| Helpful (2) |  |  |  |  |
| Moody (3) |  |  |  |  |
| Organized (4) |  |  |  |  |
| Self-confident (5) |  |  |  |  |
| Friendly (6) |  |  |  |  |
| Warm (7) |  |  |  |  |
| Worrying (8) |  |  |  |  |
| Responsible (9) |  |  |  |  |
| Forceful (10) |  |  |  |  |
| Lively (11) |  |  |  |  |
| Caring (12) |  |  |  |  |
| Nervous (13) |  |  |  |  |
| Creative (14) |  |  |  |  |
| Assertive (15) |  |  |  |  |
| Hardworking (16) |  |  |  |  |
| Imaginative (17) |  |  |  |  |
| Softhearted (18) |  |  |  |  |
| Calm (19) |  |  |  |  |
| Outspoken (20) |  |  |  |  |
| Intelligent (21) |  |  |  |  |
| Curious (22) |  |  |  |  |
| Active (23) |  |  |  |  |
| Careless (24) |  |  |  |  |
| Broad-minded (25) |  |  |  |  |
| Sympathetic (26) |  |  |  |  |
| Talkative (27) |  |  |  |  |
| Sophisticated (28) |  |  |  |  |
| Adventurous (29) |  |  |  |  |
| Dominant (30) |  |  |  |  |

End of Block: Risk and personality questions

Start of Block: Kahneman and Tversky Risk Choice 1

| 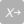 |
| --- |

Q72
Imagine there is a pandemic that is threatening people's lives. As a result of this pandemic, 600 people might die. You are working on programs that can help lessen the impact of this pandemic.


From the following two programs, please select the option that you prefer.

- Program A: If you adopt this program, 200 people will be saved (1)
- Program B: If you adopt this program, there is 33.3% probability that 600 people will be saved and a 66.7% probability that no people will be saved (2)

| Page Break |  |
| --- | --- |

| 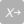 |
| --- |

Q73
Imagine there is a pandemic that is threatening people's lives. As a result of this pandemic, 600 people might die. You are working on programs that can help lessen the impact of this pandemic.


From the following two programs, please select the option that you prefer.

- Program A: If you adopt this program, 400 people will die (1)
- Program B: If you adopt this program, there is 33.3% probability that no people will die and a 66.7% probability that 600 people will die (2)

| Page Break |  |
| --- | --- |

| 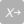 |
| --- |

Q74
Imagine there is a pandemic that is threatening people's lives. As a result of this pandemic, 600 people might die. You are working on programs that can help lessen the impact of this pandemic.


From the following two programs, please select the option that you prefer.

- Program A: If you adopt this program, 30 people will be saved (1)
- Program B: If you adopt this program, there is 5% probability that 600 people will be saved and a 95% probability that no people will be saved (2)

| Page Break |  |
| --- | --- |

| 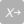 |
| --- |

Q75
Imagine there is a pandemic that is threatening people's lives. As a result of this pandemic, 600 people might die. You are working on programs that can help lessen the impact of this pandemic.


From the following two programs, please select the option that you prefer.

- Program A: If you adopt this program, 570 people will die (1)
- Program B: If you adopt this program, there is 5% probability that no people will die and a 95% probability that 600 people will die (2)

End of Block: Kahneman and Tversky Risk Choice 1

Start of Block: Choice 1 - Natural Disaster

| 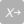 |
| --- |

Q76
Imagine there is a natural disaster that is threatening people's lives. As a result of this natural disaster, 600 people might die. You are working on programs that can help lessen the impact of this natural disaster.


From the following two programs, please select the option that you prefer.

- Program A: If you adopt this program, 200 people will be saved (1)
- Program B: If you adopt this program, there is 33.3% probability that 600 people will be saved and a 66.7% probability that no people will be saved (2)

| Page Break |  |
| --- | --- |

| 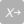 |
| --- |

Q77
Imagine there is a natural disaster that is threatening people's lives. As a result of this natural disaster, 600 people might die. You are working on programs that can help lessen the impact of this natural disaster.


From the following two programs, please select the option that you prefer.

- Program A: If you adopt this program, 400 people will die (1)
- Program B: If you adopt this program, there is 33.3% probability that no people will die and a 66.7% probability that 600 people will die (2)

| Page Break |  |
| --- | --- |

| 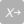 |
| --- |

Q78
Imagine there is a natural disaster that is threatening people's lives. As a result of this natural disaster, 600 people might die. You are working on programs that can help lessen the impact of this natural disaster.


From the following two programs, please select the option that you prefer.

- Program A: If you adopt this program, 30 people will be saved (1)
- Program B: If you adopt this program, there is 5% probability that 600 people will be saved and a 95% probability that no people will be saved (2)

| Page Break |  |
| --- | --- |

| 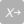 |
| --- |

Q79
Imagine there is a natural disaster that is threatening people's lives. As a result of this natural disaster, 600 people might die. You are working on programs that can help lessen the impact of this natural disaster.


From the following two programs, please select the option that you prefer.

- Program A: If you adopt this program, 570 people will die (1)
- Program B: If you adopt this program, there is 5% probability that no people will die and a 95% probability that 600 people will die (2)

End of Block: Choice 1 - Natural Disaster

Start of Block: End of Survey

| 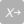 |
| --- |

Q80 Thank you for participating in our survey. Do you have any other comments that you would like to share about this survey.

- Yes (1)
- No (2)

Display This Question:

If Thank you for participating in our survey. Do you have any other comments that you would like to... = Yes

Q81 Please share your comments here:

________________________________________________________________

Q82 Browser Meta Info

Browser (1)

Version (2)

Operating System (3)

Screen Resolution (4)

Flash Version (5)

Java Support (6)

User Agent (7)

Q83 Timing

First Click (1)

Last Click (2)

Page Submit (3)

Click Count (4)

End of Block: End of Survey
